# Convolutional Neural Net Learning Can Achieve Production-Level Brain Segmentation in Structural Magnetic Resonance Imaging

**DOI:** 10.3389/fnins.2021.683426

**Published:** 2021-06-21

**Authors:** Evan Fletcher, Charles DeCarli, Audrey P. Fan, Alexander Knaack

**Affiliations:** ^1^Department of Neurology, University of California, Davis, Davis, CA, United States; ^2^Department of Biomedical Engineering, University of California, Davis, Davis, CA, United States

**Keywords:** magnetic resonance imaging, brain segmentation, deep learning, convolutional neural network, medical image processing, medical imaging data ground truth

## Abstract

Deep learning implementations using convolutional neural nets have recently demonstrated promise in many areas of medical imaging. In this article we lay out the methods by which we have achieved consistently high quality, high throughput computation of intra-cranial segmentation from whole head magnetic resonance images, an essential but typically time-consuming bottleneck for brain image analysis. We refer to this output as “production-level” because it is suitable for routine use in processing pipelines. Training and testing with an extremely large archive of structural images, our segmentation algorithm performs uniformly well over a wide variety of separate national imaging cohorts, giving Dice metric scores exceeding those of other recent deep learning brain extractions. We describe the components involved to achieve this performance, including size, variety and quality of ground truth, and appropriate neural net architecture. We demonstrate the crucial role of appropriately large and varied datasets, suggesting a less prominent role for algorithm development beyond a threshold of capability.

## Introduction

This paper focuses on the procedures, including data and training, needed for convolutional neural net learning to segment the brain cavity within structural magnetic resonance imaging (MRI). Convolutional neural nets (CNNs) (Bengio, 2009) are particularly well suited for image analysis and have recently been used in a variety of applications for medical image processing (Litjens et al., 2017; Zhang et al., 2017; Lundervold and Lundervold, 2019). Here we focus on training and testing protocols using a CNN that led to a practical, production-level application for MRI pipeline processing of brain cavity segmentation.

In this project, our goal of “production-level” processing means the capacity to perform high throughput and consistent quality image processing. High throughput means fast computation times; consistent quality means that the need for human interaction quality control (QC) is minimized. Both of these aspects are crucial to the ability for analyzing large datasets, because they currently lead to bottlenecks that limit data processing capacity. The remainder of this introduction expands upon these points.

### The Need for High Volume Data Processing

In the field of medical imaging, there is a developing consensus that diseases such as Alzheimer’s are multifactorial in origin, with subtle pathological changes beginning many years before the onset of clinical symptoms (Maillard et al., 2012; Jack et al., 2013; Knopman et al., 2013; Villemagne et al., 2013; Fletcher et al., 2014; Han et al., 2020). This suggests that a “big-data” approach will be needed to uncover and analyze the etiology and evolution of such diseases, by enabling the statistical power to detect subtle effects. A limiting factor for such analyses has historically been the lack of ability to robustly process large amounts of data, with the implicit need of harmonization (Pomponio et al., 2020), in order to generalize across inevitable sources of variability.

Deep learning holds the promise to address this limitation (LeCun et al., 2015; Akkus et al., 2017; Sun et al., 2017). However, current work on deep learning applications in many fields has highlighted the need for very large training data sets in order to make useful predictions (Sun et al., 2017). From facial recognition and image identification to game playing and language comprehension, innovative learning architectures need training on large data sets that adequately represent real-world variability of the objects to be learned. In medical image processing, the lack of adequate training data has been a long-standing problem (Akkus et al., 2017; Ker et al., 2017; Litjens et al., 2017).

### Brain Segmentation as Part of a High-Throughput Pipeline

Segmentation of the brain from the whole head (i.e., skull-stripping or brain extraction) is an essential task in neuroimaging processing pipelines. The gold standard for segmentation has historically been slice-by-slice manual human labeling, but this is slow, subject to systematic operator error, and effectively limits the achievable size of labeled data sets. In response, many standalone algorithm applications have been developed for skull-stripping, using variations on the techniques of growing a volume outward or fitting a deformable mesh to the brain surface [see (Eskildsen et al., 2012) for review]. Some of these allow user input of parameters [e.g., BET (Smith, 2002)] and some are completely automatic [e.g., ROBEX (Iglesias et al., 2011)] but in any case, performance has remained variable across different datasets (Shattuck et al., 2009; Leung et al., 2011). An alternative approach is atlas-based matching (Aljabar et al., 2009; Leung et al., 2011). This method employs a set of images (the atlases) whose brain masks have been carefully segmented *a priori*; the atlas MRIs are nonlinearly registered to the target native space MRI, followed by a voting scheme using the deformed masks to identify the most likely brain voxels within the whole head image. The brain atlas-matching approach outperformed three other extraction tools in an earlier test (Leung et al., 2011). We have found that this method is generally accurate and answers our need for segmentation on large datasets. It avoids the parameter tweaking among many available algorithms and also improves robustness across different data sets seen in other methods. However, it is computationally very expensive.

### Segmentation of the Intracranial Cavity

Another important issue arises from our protocol of segmenting not the brain but the intra-cranial cavity (ICC). Because the brain is subject to tissue loss with age, while the ICC is a relatively stable biomarker, accurate measures of ICC can be used as a reference for more precise measures of brain differences due to age or disease. Not only do accurate assessments of ICC adjust for gender-related differences in head size (DeCarli et al., 2005) but the ICC reflects maximally attained brain growth and is itself a predictor of cognitive ability in later life (Farias et al., 2012). The ICC is thus a stable benchmark that enables reliable measurements of other biomarker changes.

Intra-cranial cavity segmentation is unavailable in the other methods that aim to segment a preset brain mask but is feasible in an atlas protocol using customized atlas ICC masks. Our laboratory therefore previously adopted atlas-matching as our standard ICC segmentation step. Computation of atlas matching is intensive, however, requiring up to 15–27 CPU hours to nonlinearly register 15 atlas MRI images to the target image (Aljabar et al., 2009), followed by additional time of 15–45 min for human quality control (QC). Intensive resource use in computation and human involvement has thus remained a throughput bottleneck for our main processing pipeline.

### Previous Recent Work on Brain Segmentation

At least two approaches to CNN brain mask prediction have recently been proposed (Kleesiek et al., 2016; Salehi et al., 2017). These were shown to outperform non-CNN brain extraction applications on publicly available datasets. However, these datasets comprised relatively small numbers with limited scanner variety and variable segmentation quality: they included the LONI Probabilistic Brain Atlas Project (LPBA40) with 40 scans (Shattuck et al., 2008); the Open Access Series of Imaging Studies (OASIS) with 77 scans (Marcus et al., 2010); and the Internet Brain Segmentation Repository (IBSR) with 18 scans (Rohlfing, 2012). A dearth of high-quality medical image training data has been a limitation to many recent published methods (Akkus et al., 2017; Ker et al., 2017; Litjens et al., 2017). Such limitations stem from small quantities and lack of variability, including limited scanner variety and homogeneity of participants. This again raises the issue of large datasets – and their relative scarcity.

### The Current Project

This paper reports on our experiments to develop a CNN application for production-level, high throughput and robust identification of ICC locations in structural MRI. For machine learning to achieve production level as we have defined it, three components are required (Sun et al., 2017): a high performance computational platform, an appropriate CNN architecture, and large training datasets. We aim to fill the gaps in recent literature by demonstrating component characteristics of data, architecture and training that have achieved production level. Our laboratory has a uniquely extensive archive of over 27,000 structural MRI whole head images from multiple national imaging cohorts, representing a range of scanners and high-quality ICC masks, along with a high-performance machine learning platform (NVIDIA DGX). We hypothesized that our large archive with its variability of data could train a CNN prediction model capable of routine, fast and robust processing of large numbers of ICC segmentation masks.

## Methods

### Hardware and Software

We performed all training, testing, preprocessing, postprocessing and image prediction metric evaluations on an NVIDIA DGX Station equipped with a 20-core Intel Xeon CPU, 256 GB of system memory, and four Tesla V100 16GB GPUs.

We used the TensorFlow platform^1^ to implement and train the CNN, and to calculate the similarity metrics between predicted and ground truth ICC masks. We produced ICC mask ground truth data for training and testing via our in-house code for atlas-based brain extraction, developed as part of our image processing suite that has been detailed previously (Fletcher et al., 2018). We performed all additional processing in Python 3 using publicly available external modules including NiBabel, NumPy, SciPy, scikit-image, Pydicom, ptoml and tqdm. Result analysis was performed using Pandas or R. Visual analysis of images and masks was performed using Mango.

### Neural Network

**FIGURE 1:**
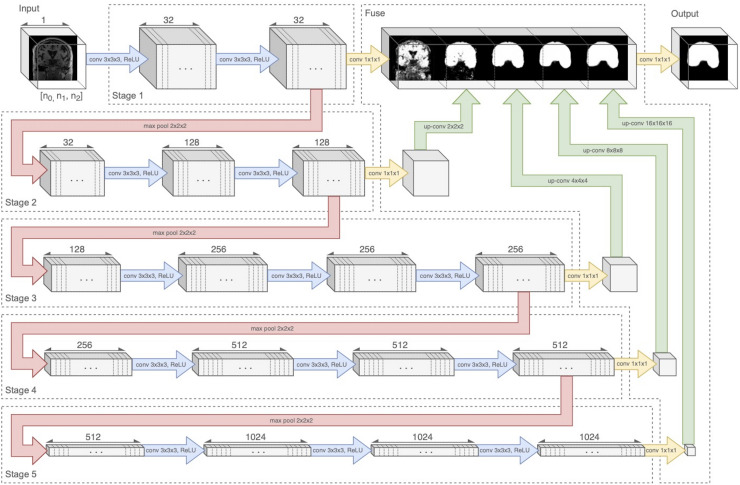
Neural network diagram tracking 3D volume through the 5-stage encoder and fuse decoder. Convolution and ReLU layers are combined in blue arrows. Upsampling is shown by green arrows. The five stages are connected by four downsample pooling layers (red arrows) that each reduce the image by a factor of 2. The final fuse layer (green arrows) upsamples each stage output to the original image resolution and fuses them to create the prediction mask. Figure modified from Fletcher and Knaack (2020).

**FIGURE 2:**
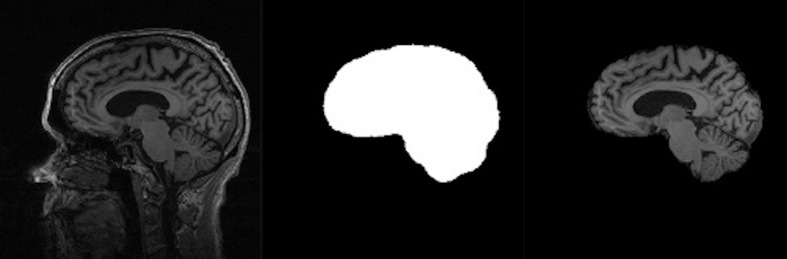
Stages in extraction of ICC from whole head volume. Left panel: whole head MRI. Middle: ICC mask. Right: Resulting ICC volume image. Note the ICC boundary extending beyond the cortical edge to the dura mater boundary. Figure modified from Fletcher and Knaack (2020).

Our architecture was an end-to-end volumetric 3D CNN adapted from a network that was previously used for vascular boundary segmentation in 3D computed tomography scans (Merkow et al., 2016). One of our experiments was to determine the effects on prediction performance of varying the number of stages in the CNN. As an illustration of the architectural framework for our CNN approach, our best version is outlined in Table 1 and illustrated in Figure 1. It takes as input a whole-head 3D structural MRI volume and outputs maps estimating the relative strength (from 0 to 1) of brain membership for each voxel location in the MRI. Binary brain segmentation masks are derived by thresholding the probability maps at an empirically derived value of 0.34 to maximize the median Dice match score and minimize the interquartile variability ranges of the match scores. In the architecture reported in Table 1 and Figure 1, the encoder consists of 13 convolutional/ReLU layers divided into 5 stages of decreasing image resolution. Successive stages are connected, and spatial dimensions are reduced, via 4 max-pooling layers. The decoder consists of 6 convolutional layers. To create a fused output mask prediction, the output images from each stage is reduced using a 1 × 1 × 1 convolutional layer (yellow arrows of Figure 1), then upsampled to the original input dimensions using trilinear interpolation in the form of a non-learning convolution transpose layer. One last 1 × 1 × 1 convolutional layer then fuses the multi-resolution outputs from each stage, which is bound to the range [0,1] using a sigmoid activation layer. The CNN output is thus a number in the range [0,1] for every MRI voxel; we thresholded the output as described above to obtain the prediction mask (Figure 2). Experiments for this paper involved altering the number of stages in this basic design, to test 2,3,4 or 5 stages.

**TABLE 1:** CNN architectural detail.

Stage	Layer count	Filter size	Layer filter count
1	2	3 × 3 × 3	32
2	2	3 × 3 × 3	128
3	3	3 × 3 × 3	256
4	3	3 × 3 × 3	512
5	3	3 × 3 × 3	1024

*All architectures share the same scheme illustrated here for the 5-stage best-performing model.*

### Extracted Intra-Cranial Cavity Mask Definition

Most skull-strip algorithms aim at segmenting the brain at its tissue boundary (i.e., with cerebrospinal fluid) in the whole head image. That extracted mask thus excludes any tissue and space outside the brain. However, our protocol extracts the ICC head space beyond the brain out to the dura mater anteriorly, dorsally and posteriorly, as illustrated in Figure 2. The dura/CSF boundary is easily visible as a line between gray intensity (dura) and CSF (black) (Figure 2, left panel). This segmentation has enabled us to test for longitudinal consistency as measured by the volumetric segmentation of ICCs from sequential scans of a single individual, since the ICC should not change over time.

### Ground Truth Data, Training and Testing

#### Data Set Composition

For training and testing, we used 10,378 structural T1-weighted MRI brain scans selected from our archive of over 27,000 scan sessions, representing data from 17 national imaging studies, from which training sets were drawn with up to 11 imaging cohorts in a training set. The available imaging cohorts and data set sizes are detailed in Table 2. Training and testing sets selected from this pool were varied to test the influence of the two factors of training set size and variety of imaging cohorts for output predictions. No participant with serial scans had one scan in the training set and another in the test set. The available diversity of imaging cohorts by subject demographics and MRI acquisitions allowed us to experiment with the best combination for robust segmentation in the face of image variability across sites.

**TABLE 2:** Imaging sources for our ground truth data.

Cohort	Cohort numbers			Cohort characteristics			
	Train	Eval	Test	Scanner (3T %)	Scanner Models (N)	Sex (M %)	Avg Age
A	102	34	34	1.00	1	0.27	93.4
B	381	50	59	0.50	7	0.22	76.0
C	2696	337	26	0.15	16	0.07	74.0
D	244	39	37	0.00	3	0.41	58.0
F	209	37	35	0.00	3	0.40	79.5
G	80	33	33	1.00	1	0.36	71.5
H	2849	357	348	0.23	11	0.46	58.0
I	0	0	6	0.50	2	0.00	N/A
J	74	33	9	0.09	2	0.05	49.3
K	0	0	50	1.00	1	0.02	N/A
L	0	0	10	1.00	1	0.40	64.5
M	0	0	73	0.99	2	0.63	74.4
N	0	0	2	1.00	1	0.50	94.0
O	1182	147	119	0.41	17	0.17	63.7
P	157	37	37	1.00	3	0.04	50.9
Q	333	48	47	0.94	4	0.51	70.3
R	0	0	14	0.00	2	0.14	N/A
Totals	8307	1152	939	0.33	28	0.29	63.3

*All ground truth data was generated by our atlas-based plus human QC as described in the text. Basic scanner characteristics are number of different scanner models and percentages of 1.5T and 3T field strengths; percentage for 1.5T is 1 minus the 3T values shown. Thus, for example, 0 for cohort R means all its scans had 1.5T field strength; similarly, 1.0 for cohort A indicates all scanners at 3T. Similar presentation for demographic breakdown by sex. Average ages are shown in cohorts where available.*

#### Atlas-Based Mask Generation

Each structural MRI had a segmented ICC mask created by an automated multi-atlas segmentation procedure (Aljabar et al., 2009) followed by human quality control. Briefly, the multi-atlas segmentation consisted of nonlinear warps of 15 template brain images, in which the ICC has been carefully delineated by human operators, onto a target whole-head MRI. The deformed template ICC masks were overlaid in the target native space and a voting scheme is conducted to ascertain a consensus of ICC locations within the target image. Final cleanup to remove inevitable mistakes was performed by human operators using a slice-based visualization tool.

#### Human Quality Control

Human QC is an indispensable final step of any computational image processing method. As such, it accounts for a significant portion of the resource use, in time and expense, to achieve usable data. In ICC mask production, human QC is necessary to inspect, and where required, manually remove misestimations of mask boundaries, all on a slice-by-slice basis using a slice visualization tool. In our experiments, all ground truth (GT) data included a final human QC step. With our atlas-based mask segmentations, this QC takes from 15 to 45 min per image.

**FIGURE 3:**
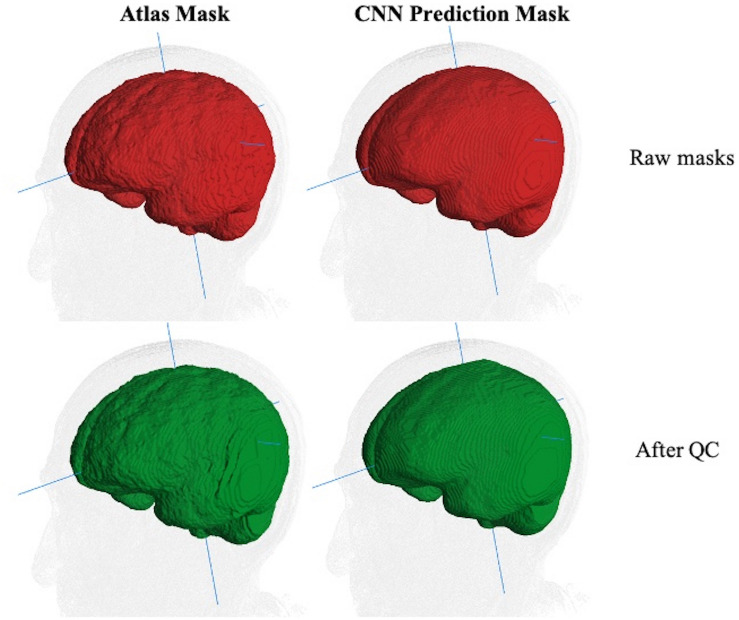
Comparison of ICC mask quality before and after human QC. The same MRI image with an atlas-based GT (left column) and CNN prediction mask (right). Top row (red): raw masks before QC. Bottom row (green): masks after human QC. The edge-based variability is a source of random noise in ground truth. Less variability is introduced for human QC of CNN predictions than for atlas-based masks.

However, human inconsistency across slices introduces edge unevenness that adds random noise. The effects of slice-based edge inconsistencies compared to CNN predictions are seen in Figure 3, which shows two estimations of the ICC mask for the same subject. The left panel shows the effects of human QC on an atlas-based mask. The right panel shows the same for a CNN prediction. By the CNN’s capacity to generalize, the CNN estimates tend to be inherently smoother than the atlas based, thereby potentially diminishing introduced noise from human QC. We therefore hypothesized that CNN outputs combined with human QC would benefit not only by drastically reduced computation time but also reduced human interaction time.

In sum, GT for all training, test and validation sets consisted of atlas-based ICC masks completed by human QC. Each of the three sets was completely disjoint from the others, having no images from any participant in more than one set.

#### Training

Network training was deeply supervised, with loss function penalties calculated at each stage as well as the final fused prediction. Training example pairs were sampled one at a time in round-robin fashion by cohort. Individual cohort sets were continually cycled over successive training iterations to maintain influence until a fixed number of training steps had been completed. Specifically, at each training cycle all cohorts are represented via round-robin selection. This means that over all cycles of the training, images from smaller cohorts are seen in more of the cycles than images from larger cohorts. Training was completed in a range between 12 and 44 hours depending on the number of cohorts and CNN architecture, and using the following hyperparameters:

• Loss function: Summed cross-entropy of each stage and the fused prediction compared to ground truth (Merkow et al., 2016).
• Optimization: Exponential moving average of a stochastic gradient descent with Nesterov momentum (Sutskever et al., 2013). All gradients were clipped so that their L2-norm was less than or equal to 4 before they were applied by the optimizer.
• Learning rate: 10^–2^
• Momentum: 0.9
• Moving average decay: 0.999
• Batch size: 1
• Steps: 30,000

#### Image Pre- and Post-processing

Pre-processed images were auto-cropped and padded to achieve minimal background while conforming to lattice dimensions divisible by 2^*N*–1^, with N corresponding to the number of image resolution stages in the CNN (see Figure 1 where *N* = 5). Intensities were normalized to unit SD with zero mean after the image was cropped, but before it was zero padded.

To preserve the original volumes and save space, pre-processing steps are calculated non-destructively, and applied immediately before neural network processing.

To produce CNN based ICC masks, the CNN prediction maps for voxels to be included were binarized using a threshold of *p* > 0.34, empirically determined to produce the best mask based on quantitative results from our performance metrics. This threshold was therefore not derived from training, but rather a postprocessing step used to maximize the overall matching performance with test sets.

### Metrics for Evaluating the ICC Mask Predictions

We evaluated the performance and quality of the CNN brain mask predictions using three comparison measures: *model generalization, model consistency* and *resource efficiency*.

A. Model generalization refers to the ability of the trained neural net to match ground truth masks of the test samples across a variety of imaging cohorts. This is important because imaging cohorts vary by characteristics of scanner and participants, and we want to achieve consistently good matches regardless of cohort. We used the Dice similarity coefficient (DSC) (Dice, 1945) for match quality between each CNN mask and its corresponding ground truth mask. DSC is commonly used measure of mask outputs and enabled a direct comparison of our results to mask performance with other methods (Shattuck et al., 2009; Eskildsen et al., 2012; Kleesiek et al., 2016; Salehi et al., 2017) The DSC is defined as follows:

$$DSC=\frac{2\left|A\cap B\right|}{\left|A\right|+\left|B\right|}$$

where *A* is the set of predicted voxels in the CNN mask and *B* is the set of voxels in the GT mask.

In a randomly selected smaller dataset (N = 400) we also evaluated model performance using the Hausdorff distance (Huttenlocher et al., 1992) of prediction mask to GT, and false negative and positive labeling. These three measures provided complementary information about CNN model performance. In our context, the Hausdorff distance (HD) measures the extent to which each point in the CNN segmentation mask is close to some point of the GT mask, and vice versa. HD is thus a measure of similarity between two masks. It is defined as follows:

HD=max(h(A,B),h(B,A))

where,

$$h\left(A,B\right)=\max_{a\in A}\min_{b\in B}||a-b||$$

and ||*|| indicates the Euclidean distance between points in masks A and B. Because HD depends on maximal distances, it could be large even though the masks almost entirely coincide, if a point of one mask lies a great distance outside the other. For our purposes of evaluating a production algorithm, we therefore also computed how much of the target mask was “missed” or failed to be labeled by the CNN prediction; this is a measure of how much infill would have to occur during human QC. Likewise, we computed the percentage of the CNN mask lying outside the target GT, thereby giving a measure of how much removal would have to be performed by human QC.

B. Model consistency is the ability to generate ICC masks for longitudinal same-subject repeated scans that are close in volume. Unlike brain volume, estimated ICC volumes are expected to be unchanged over repeated scans. We used the maximum volumetric differences over all scans of a subject to assess this consistency. Consistency tests are a valuable metric of prediction quality and robustness, given that absolutely accurate GT is out of reach.

C. Resource efficiency encompasses the two aspects of computational and human resource time. Our atlas-based brain mask computations require about 15–27 CPU hours of computation followed by an average of 15–45 min of human QC. We compare the corresponding times for computation and human QC of the CNN masks.

### Summary of Experiments Varying Training and Testing Protocols and CNN Architecture

#### Effects on Performance of Number of Cohorts vs. Raw Dataset Size

We fixed the number of CNN stages at five and evaluated the effect on output quality by varying the overall number of subjects in the training set and the number of separate imaging cohorts. This tested the relative importance of sheer dataset sizes vs. number of imaging cohorts, where each cohort may embody a constellation of imaging characteristics – scanner field strength, manufacturer, model and image acquisition parameters, along with differences in subject population age and clinical diagnoses – that distinguishes it from the others. Cohort scanner and demographic characteristics are presented in Table 2.

**FIGURE 4:**
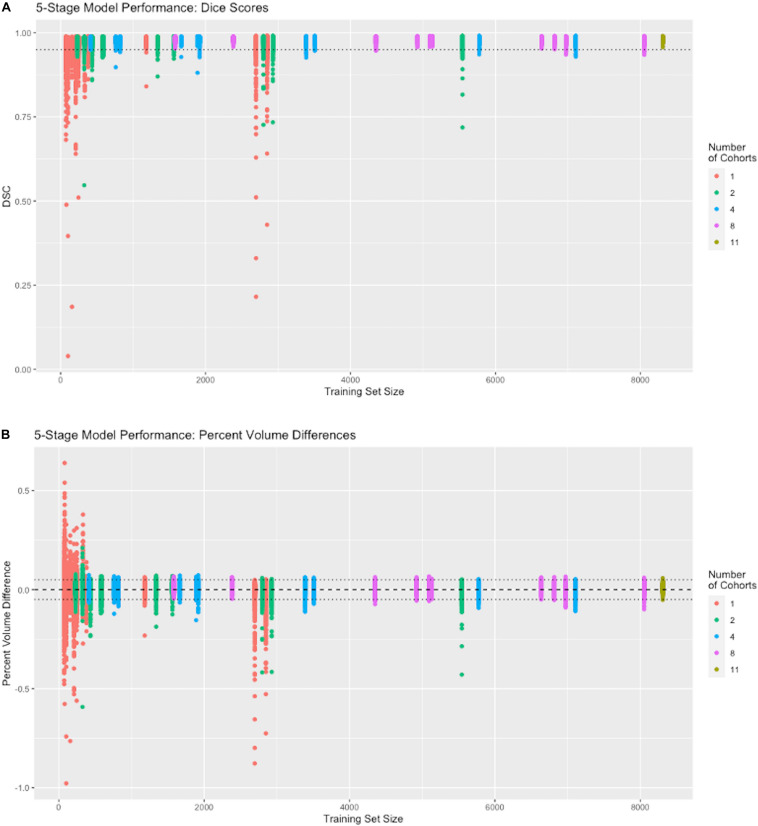
Performance vs. number of cohorts in training. The *x*-axis shows the number of samples in each individual training session (*N* = 74 to 8309). The number of cohorts (1, 2, 4, 8, and 11) in a training set is labeled by color (see legend). Following the protocol in Section “Effects on Performance of Number of Cohorts vs. Raw Dataset Size,” each colored vertical line plots the distribution of DSC matches to 939 test subjects from a CNN model corresponding to one training set. For example, there are 11 red vertical lines, one for each of our 1-cohort trainings, though some are hard to distinguish because of overlap due to similar training set size numbers shown in Table 2. There is one distribution for the one 11-cohort training. For all others, there are 10 vertical lines of each color. **(A)** Dice similarity performance against test set ground truth based on training sets of variable cohort diversity. Dotted line indicates DSC = 0.95. **(B)** Volume difference values (CNN prediction – GT mask volume) as percentage of GT mask volume. Dotted lines indicate ±0.05, i.e., ±5 percent.

To run the experiments reported in Figure 4, we performed 42 separate trainings of our 5-stage CNN architecture, using the 11 cohorts with nonzero Training set numbers in Table 2. Each training resulted in a different CNN model that was then tested on 939 subjects in the cohorts with nonzero Test numbers of the same table. The cohort representation in each training set was determined as follows. For 1-cohort trainings, we used each of the 11 cohorts in turn, giving 11 different 1-cohort CNN training models. For all 11 cohorts, there was only one training. For each of the other numbers of cohorts, the number of possible trainings (11 choose N, where *N* = 2,4,8 cohorts), was impossible to explore fully given limited resources, so for each N we chose to do 10 training models, selected so that each cohort was represented in at least one N-tuple among the 10 trainings at that N. Each training model produced a single output of 939 DSC match plots with the test set, color coded by N and positioned along the *x*-axis by raw size of training set, in Figure 4.

#### Effects on Performance of Number of Stages in the CNN Architecture

Using the best combination of imaging study variety and number of subjects from the first test, we examined differences that may be produced by varying the number of CNN stages, from 2 to 5. This tested the importance of CNN architecture parameters on mask prediction quality.

#### Performance Evaluation Using Additional Similarity Metrics

We evaluated our best model CNN performance on a randomly selected subset (*N* = 400) using three additional measures: Hausdorff distance, and extents of false negative and positive labeling, providing complementary measures (maximal and average-based) of mask shapes.

#### CNN Prediction Consistency for Serial Same-Subject Scans

This tested the constancy of the ICC segmentations in single subjects, which ideally should not change over time. Although it is impossible to achieve ground truth that perfectly represents extracted volumes within the skull, we can nevertheless test whether the CNN produces consistent ICC volume predictions over time.

## Results

### Composition of Ground Truth Data

Table 2 shows the imaging cohorts and partitioning of each cohort into training, evaluation and test sets. We used ground truth data from 17 imaging cohorts (left column of Table 2) with ICC masks generated using atlas-based segmentation. We used a combined sum of 10,398 images divided among training, evaluation and test sets as shown. These data cover the entire breadth and depth of scanner characteristics including differences in MR vendors and models and field strengths that vary between 1.5 to 3T. There is similar variability in demographic characteristics of age and sex, factors which can change characteristics of the acquired MRI due to differing age-related atrophy levels and gender head size. We note relatively young ages of 48 years for cohort J; 58 years for cohorts D and H; middle-old (in the 70 s) for B, C, F, G; and oldest-old (above 90 years) for A and N.

### Results of Experiments

Except where otherwise noted, test results were generated from the full test set described in Table 2.

#### Effects on Performance of Cohort Counts vs. Training Set Size

Using a five-stage CNN (see Figure 1) we conducted individual training sessions from GT incorporating 1, 2, 4, 8 and the full complement of 11 imaging cohorts. The data set was our full set of atlas-based GT that is described in Table 2. These experiments tested the effects of number of cohorts represented (i.e., its “diversity”) and number of subjects in a training set on performance measures. Results are displayed in Figure 4, showing the performance (measured as dispersion of Dice similarity scores in A and percent volume differences from GT in B) as a function of these variables. Summary statistics appear in Table 3. All test results were generated from the test set described in Table 2.

**TABLE 3:** Summary statistics for performance from training runs using 1, 2, 4, 8, or 11 cohorts.

Number of cohorts	DSC		
	Median	Mean	SD
1	0.977	0.969	0.032
2	0.982	0.979	0.012
4	0.983	0.982	0.006
8	0.984	0.983	0.005
11	0.985	0.984	0.004

*Entries display the median, mean and SD of DSC test set matches over all runs for each stated number of cohorts. CNN model: 5-stage.*

Although both the raw size of training sets and number of imaging cohorts in each training affected outcome performance, these results suggest that the number of represented cohorts, or *diversity* of the training set, is a stronger determinant for robustness of performance than the raw numbers of subjects. For example, with one training cohort the dispersion of DSC scores is high regardless of the number of subjects in the training set (red plots in Figure 4, training sizes *N* = 74 to 2849). Training with over 2000 subjects did not obtain a better DSC dispersion than using only 74 subjects. Conversely, for trainings with 8 cohorts, the dispersion of DSC predictions was uniformly low regardless of the number of training examples (magenta plots in Figure 4A, training *N* = 1582 to 8053), and very close to the performance achieved by using all 11 cohorts (training *N* = 8309). Note in particular that using 8 cohorts (magenta) with training set sizes just below or above 2000 show appreciably lower dispersion than for nearby plots of 2 (green) or 4 (blue) cohorts with similar sized training sets; and not different than those for much larger training sets having 8 cohorts. The summary results of Table 3 suggest a stabilized median DSC performance at 4 cohorts and above, with median DSC above 0.98. However, Figure 4A indicates longer low outlier “tails” of DSC values among the runs trained on 4 cohorts, and Table 3 shows a roughly 50% higher SD for 4 cohorts, suggesting that stabilization occurred at 8 cohorts. Measures of volumetric mismatch (Figure 4B) show differences approaching 50% of GT targets for trainings with one or two cohorts, with differences declining to near 5% with 8 cohorts.

#### Performance vs. CNN Stage Count

**FIGURE 5:**
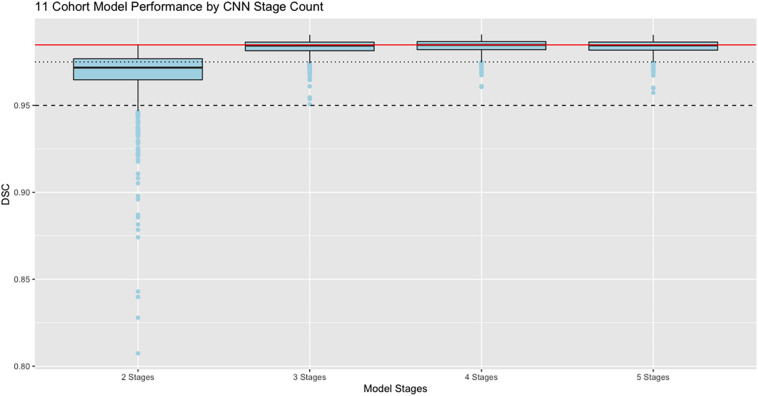
Performance vs. number of stages. Dice similarity performance against test set ground truth (*N* = 939) based on number of stages in the CNN, using the full 11 cohort training set. Red line is the median value 0.9848 achieved by the 4-stage architecture to allow easy comparison with the other models. Dashed line indicates DSC = 0.95; dotted line is DSC = 0.975. Boxes show lowest 25%, median, and 75% thresholds.

Our next results report the effects of varying the number of stages in the CNN architecture, using the full training set of 11 cohorts with 8307 training examples whose performance is at the far right of Figure 4. Results are displayed in Figure 5, with box and whisker plots showing the dispersion of Dice similarity scores as a function of number of stages.

In these results, the model performance of Dice scores vs. test set ground truth achieves a peak at DSC = 0.9848 using 4 stages, with highest median value and lowest dispersion of the prediction scores. The mean performance was DSC = 0.984 using 5 stages.

#### Measures of Hausdorff Distance and Percent Mismatches

**FIGURE 6:**
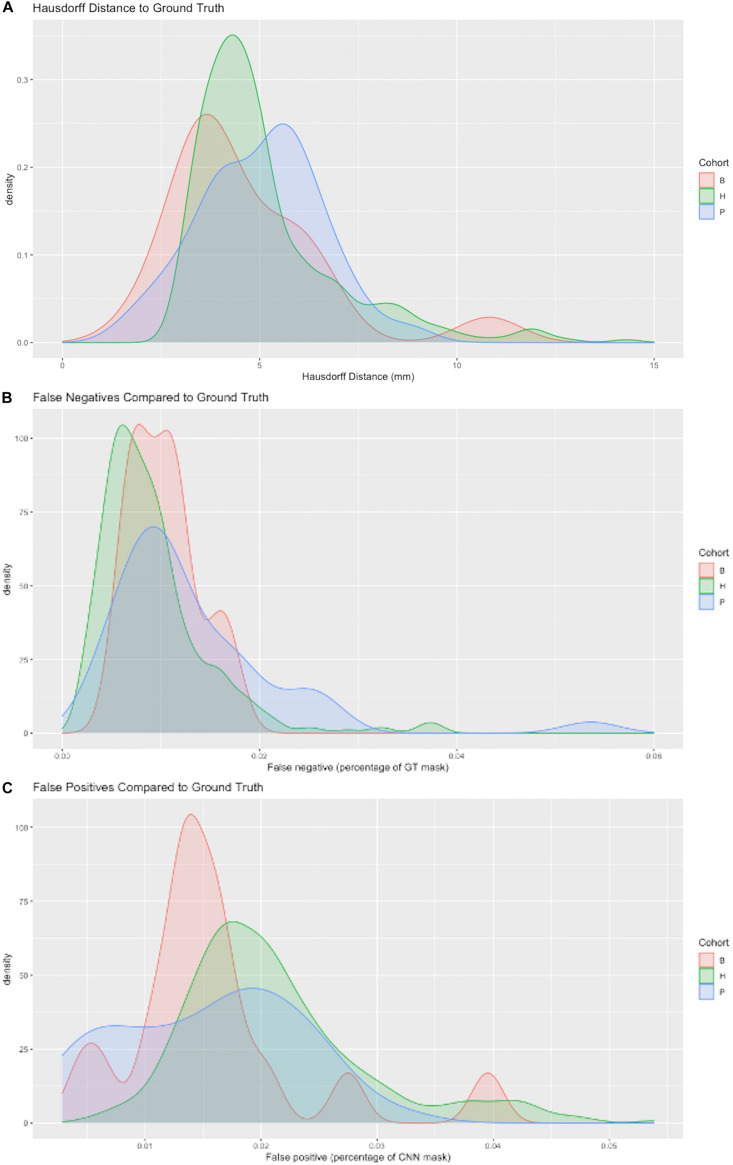
Three metrics of model performance. In a randomly selected subset of the data, the best performing model was evaluated against GT masks using **(A)** Hausdorff distance **(B)** False negatives as a percentage of GT mask volume missed by CNN prediction and **(C)** False positives as a percentage of the CNN prediction incorrectly labeling areas outside GT mask. Results are depicted by probability density functions for each of three cohorts.

Figure 6 displays probability density functions for a randomly selected subset (*N* = 400) of the test set (Table 2) containing three cohorts. The model tested was our top performing model that used 11 cohorts for training and 5 stage CNN architecture. Figure 6A shows HD measure performance. The modes of HD are from 3 to 6 mm, varying by cohort. For cohort H there were three outliers with HD > 25, not shown in this graph to avoid scale compression. Visual examination of the CNN masks showed close correspondence to the GT, except for small regions in GT not included and at large distances from the CNN mask. The percentage of false negatives (Figure 6B) and false positives (Figure 6C) did not have any outliers, confirming that the areas of the HD outliers were very small. Modes of false negatives lie between 0 and 2%, and false positives between 1 and 3%.

#### Testing for Longitudinal Consistency

**FIGURE 7:**
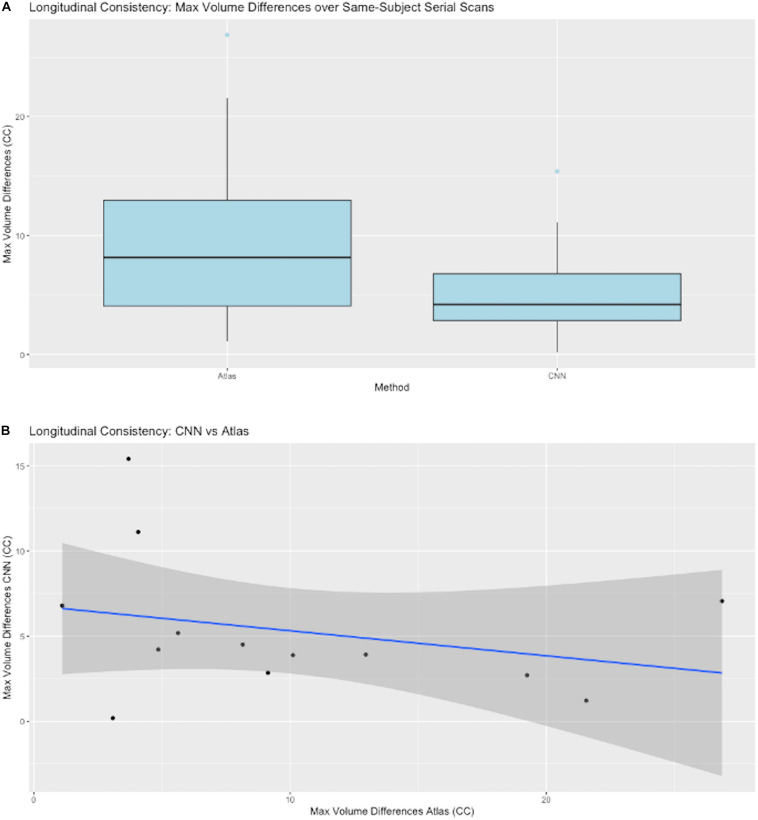
CNN model performance on same-subject repeated scans. **(A)** Distributions of maximum volume differences in estimated same-subject ICC masks. Left entry: the max volume differences for ground truth sets using atlas-based masks. Right: the same performance metric for CNN prediction masks. **(B)** Plots of CNN vs. atlas estimated max differences by subject. The straight line is a regression fit of the plots, suggesting that average CNN-predicted differences are roughly constant while corresponding atlas-based predictions vary over a large range.

A key test of segmentation performance is the consistency of ICC segmentation for serial scans of the same subject, where little change is expected. Figure 7A shows the dispersion of raw volume differences in cubic centimeters for ICC segmentations of successive same-subject MRI scans. We tested performance on 13 subjects for which longitudinal data was available and not used in CNN model training. CNN mask volume estimates differed by less than those for atlas-based masks. The medians of the maximum same-subject volumetric differences were on the order of 6 cm^3^ for CNN-based GT. Figure 7B plots CNN-estimated max differences against those of atlas GT.

#### Generalization Across Imaging Cohorts

**FIGURE 8:**
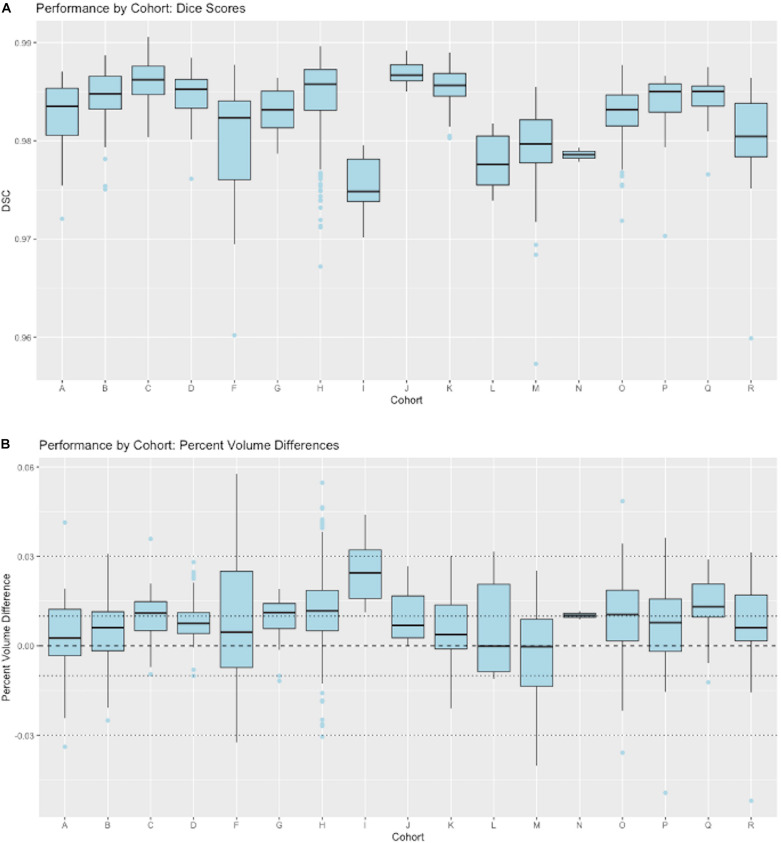
CNN performance by imaging cohort. These show matching scores for the best performing model in Figure 4 (5-stage, 11 cohort). **(A)** Box plots of DSC distributions. **(B)** Same for percentage volume mismatches (predicted volume – GT volume)/GT volume.

Figure 8 presents granular results by cohort for distributions of Dice similarity coefficients in individual imaging cohorts. Test results come from the full test set. Aggregate results of these performances across our ensembles of cohorts were presented in Figure 4. Here, we illustrate the performance *by cohort* for a single model, the 5-stage architecture trained on 11 cohorts. Figure 8A displays boxplots of DSC scores by cohort; Figure 8B shows distributions of percent volume differences with GT. It is noteworthy that these show a trend toward volume overestimation of around 1% on average by the CNN method.

#### Resource Usage

With the 5-stage model trained on 11 cohorts, we predicted ICC masks in about 10 s per image, with an additional average 10 min of human QC. The computer and human resource use for CNN prediction contrasts with a range of 15–27 CPU hours followed by 15–45 min of human QC for atlas-based ICC masks.

## Discussion

### Summary of Findings

This paper has focused on the aspects of architecture, data and training involved in high-throughput production, rather than on innovation of new CNN approaches. The experiments described here have outlined an effective CNN configuration and training to attain production-level performance of ICC segmentation. For the task of segmenting intra-cranial cavities, we report the following findings: (1) A variety of imaging cohorts is more important than sheer numbers of training subjects, with results reaching close to a plateau at 4 cohorts in the training sets for summary measures (Table 3), although training with 8 cohorts achieves a more robust performance in which DSC and volumetric difference dispersions indicated that every trained model performed very similarly to the use of 11 training set cohorts (Figure 4 and Table 3). Thus, training across multiple cohorts above a threshold number confers an excellent ability to generalize successful predictive power (Figure 8) that is insensitive to variability from the broad range of MR machines, models and field strengths studied here. (2) Architectures with four or five stages both perform very well, and both are markedly better than two stages (Figure 5). This suggests that above a threshold number of stages, performance is consistently good and is not sensitive to variations in CNN architecture. (3) Performance for three image similarity measures (Figure 6) showed generally low HD distance and very small percentage of false positive and negative mismatches, suggesting good shape resemblance (HD) and reduced human cleanup times to fill in or erase incorrect labeling. (4) The CNN predictions attain excellent longitudinal consistency that exceeds the performance of atlas-based ground truth sets (Figure 7). The CNN predictions attained median volumetric ICC differences over same-subject scans of about 4 cm^3^ (Figure 7) or roughly 0.4% in a putative brain of 1000 cm^3^, suggesting that analyses based on these outputs could offer excellent statistical power.

### Comparisons With Previous Approaches

Our performance findings appear promising when compared to recent other CNN applications for brain extraction. Over large evaluation sets containing a variety of imaging cohorts with variable MR machinery (Table 2) our best model median Dice scores were 0.9846 with tiny mean variance (Table 3 and Figure 5). For comparison, the auto-context CNN brain extraction scores (Salehi et al., 2017) were 0.977 and 0.9762 over the LPBA40 and OASIS datasets, respectively. Meanwhile the other recent CNN brain extraction algorithm (Kleesiek et al., 2016) reported a combined Dice score over IBSR, LPBA40 and OASIS of 0.958. These articles also compared their CNN segmentation against other, standalone brain extraction tools on the same datasets and found their CNN DSC scores to be the best. A direct comparison between our results and the other two CNN approaches, as well as with the standalone tools to which those were compared, was not possible because we segmented different regions: the ICC rather than a brain mask. Our DSC scores therefore measure matches with ICC ground truth, whereas the DSC scores in other studies reflected brain mask GT. However, these studies provide a context suggesting that our scores are appreciably better over much larger datasets that have undergone rigorous human quality control. This highlights the need for large and variable GT data sets to attain robust production quality. In sum, while those studies demonstrated direct superiority over standalone tools on small, relatively homogeneous data, they did not show that their trained models were ready to perform robustly over large and highly variable imaging datasets.

### The Role of Large Datasets

The scarcity of very large medical imaging data sets has spurred recent approaches toward developing innovative CNN architectures able to generalize from small samples. These have included fully 3D image recognition involving versions of U-Net architectures (Kleesiek et al., 2016; Salehi et al., 2017) for brain segmentation discussed in this paper. But other approaches, for example to segment brain white matter hyperintensities, have proposed 2D patch inputs (Ghafoorian et al., 2017b) or 3D approaches (Ghafoorian et al., 2017a), each also accompanied by input location information; or multi-modal image inputs (Moeskops et al., 2017). Those approaches perform well. However, the findings of our project suggest that those may be algorithmic “workarounds” necessitated by data unavailability, and that if large amounts of high-quality GT are available, then a useful, high throughput production pipeline is attainable. This paper thus fills a gap in the literature of techniques for MRI image processing (Akkus et al., 2017; Litjens et al., 2017; Lundervold and Lundervold, 2019). To our knowledge, this is the first report of systematic experiments documenting the variability of datasets, a CNN architecture and training protocols that in combination have achieved production levels of brain extraction, an indispensable pipeline step for MRI processing.

### The Interplay Between Data Heterogeneity and Algorithmic Complexity

Results of our study suggest an inverse relationship between CNN complexity and data heterogeneity that together can attain consistent, high-quality predictions. Figures 4A,B indicated that cohort diversity was more important than raw training size for generating tight distributions of predictions with our 5-stage CNN model. Figure 5 suggested that CNN models of 3, 4 and 5 stages all performed comparably based on 11-cohort training. Combined, they suggest the hypothesis that high-quality predictions from training on heterogeneous data sets are relatively insensitive to variations in CNN architecture, as long as this is beyond a threshold level of complexity. Explorations of this hypothesis could include using the 42-training protocol for our CNN architectures having less than five stages in order to test whether lower-stage architectures generate a pattern of prediction matches similar to Figure 4. It could also involve similar experiments using publicly available CNN architectures trained on our data. Because of current limitations of time and resources, we were not able to carry out such experiments for the current project, but this would be a promising line for future research.

### Limitations

A main limitation of our study is the inability to directly compare the performance of our trained CNN models against other CNN or non-machine learning algorithms, because currently these are aimed at segmenting the brain itself rather than ICC. However, by comparing our similarity metrics with those of previous approaches and showing reliability of ICC volumes over longitudinal scans, we have strong indirect evidence that our approach provides more robust outputs than previous methods. This hypothesis will need to be verified by direct comparisons in the future. But such a comparison will only be relevant when other CNN architectures are trained to segment the ICC mask using comparably small-sized data sets as have been reported, while ours is trained with a large dataset such as described here. This would be a direct test of the finding that datasets of sufficient size and variability make a crucial difference regardless of the CNN architecture employed, beyond some level of complexity (in our experiments, beyond 4 layers). This leads to the second limitation, which is the atypical size itself of the dataset we used. We recognize that many labs do not have access to large datasets, and this may seem to undermine the apparent relevance of our work. Two responses are pertinent here. The first is that given the rarity of large datasets, novel approaches for use with small training cohorts are appropriate and necessary. But this is not the problem we aimed to address. Instead, we aimed to show that with adequate data and training (which does exist although rare) we can settle the question of a usable, production-level pipeline for data processing. The second response is that despite lack of training data, the larger community may still benefit by using our CNN model on their own images. And we may benefit by evolving our training set with images upon which the model fails in wider use.

### Model Availability

Our CNN model will be available by request. Because we are unable to release the entirety of our data set, and efficient training also requires significant hardware investment, we will make available our current best performing model for those interested in generating their own ICC segmentations on T1w whole head MRIs. Prediction times will vary depending on platform, but CPU-based predictions are on the order of 1 min. We invite this interest in order to improve the model by finding and incorporating images on which it performs poorly, as we work toward a more generalized stable model for wide public release.

## Conclusion

This article has put forward two main points, First, we have achieved a robust, practical method of ICC extraction, bringing the goal of very large MRI dataset production within reach. Based on our findings and experience in our laboratory, we have deployed the segmentation described here as part of our routine production, replacing our previous atlas-based method. Second, our results suggest that for this task, the importance of sufficient data heterogeneity outweighs CNN algorithm complexity, at least beyond a threshold level. This hypothesis merits further research.

## Data Availability Statement

The trained CNN model using 5 stages and trained on 11 cohorts supporting the conclusions of this article will be made available by the authors, without undue reservation.

## Author Contributions

EF in consultation with AK and CD, researched CNN architectures and designed CNN prediction experiments, helped gather and prepare the data, and wrote the manuscript. AK primary designer, programmer and implementer of CNN architectures and experiments. Consulted with EF and CD on relevant experiments for pipeline image processing, collected all data and created all results figures and tables, and edited the manuscript. AF edited the manuscript and provided feedback on clarity and relevance of experiments. CD consulted with AK and EF on relevant experiments, was either the principal investigator or site investigator of all the neuroimaging grants that acquired the test data, provided context for experiments relevant to production-level performance, and edited the manuscript. All authors contributed to the article and approved the submitted version.

## Conflict of Interest

The authors declare that the research was conducted in the absence of any commercial or financial relationships that could be construed as a potential conflict of interest.

## Footnotes

1 https://www.tensorflow.org/
